# Trustable Environmental Monitoring by Means of Sensors Networks on Swarming Autonomous Marine Vessels and Distributed Ledger Technology

**DOI:** 10.3389/frobt.2020.00070

**Published:** 2020-05-28

**Authors:** Ivan Berman, Enrica Zereik, Aleksandr Kapitonov, Fabio Bonsignorio, Alisher Khassanov, Aziza Oripova, Sergei Lonshakov, Vitaly Bulatov

**Affiliations:** ^1^Faculty of Control Systems and Robotics, ITMO University, Saint Petersburg, Russia; ^2^Institute of Marine Engineering, Italian National Research Council, Genova, Italy; ^3^Heron Robots, Genova, Italy; ^4^Airalab, Tolyatti, Russia; ^5^Faculty of Food Biotechnologies and Engineering, ITMO University, Saint Petersburg, Russia; ^6^M2M Economy, Inc. (“Merklebot”), San Francisco, CA, United States

**Keywords:** unmanned surface vessel, robonomics, environmental monitoring, belief space planning, boids model, blockchain, mobile sensors, water quality

## Abstract

The article describes a highly trustable environmental monitoring system employing a small scalable swarm of small-sized marine vessels equipped with compact sensors and intended for the monitoring of water resources and infrastructures. The technological foundation of the process which guarantees that any third party can not alter the samples taken by the robot swarm is based on the Robonomics platform. This platform provides encrypted decentralized technologies based on distributed ledger tools, and market mechanisms for organizing the work of heterogeneous multi-vendor cyber-physical systems when automated economical transactions are needed. A small swarm of robots follows the autonomous ship, which is in charge of maintaining the secure transactions. The swarm implements a version of Reynolds' Boids model based on the Belief Space Planning approach. The main contributions of our work consist of: (1) the deployment of a secure sample certification and logging platform based on the blockchain with a small-sized swarm of autonomous vessels performing maneuvers to measure chemical parameters of water in automatic mode; (2) the coordination of a leader-follower framework for the small platoon of robots by means of a Reynolds' Boids model based on a Belief Space Planning approach. In addition, the article describes the process of measuring the chemical parameters of water by using sensors located on the vessels. Both technology testing on experimental vessel and environmental measurements are detailed. The results have been obtained through real world experiments of an autonomous vessel, which was integrated as the “leader” into a mixed reality simulation of a swarm of simulated smaller vessels.The design of the experimental vessel physically deployed in the Volga river to demonstrate the practical viability of the proposed methods is shortly described.

## 1. Introduction

Water resources are crucial for the maintenance of human life. Natural water is an exhaustible, partially renewable resource. Fresh water is used both for drinking water supply and in industry, agriculture, transport—in almost all human activities. Depending on the desired usage, the requirements for water chemical composition and physical properties may be different.

Worldwide water consumption has been increasing at about 1% per year since the 1980s (World Water Assessment Programme, 2019), and it is due to the growing demand for water in developing countries, where population is still increasing, to the acceleration of socio-economic development and to the fact that consumption patterns began to evolve (Alharsha et al., 2018; World Water Assessment Programme, 2019) in a similar way to those of old and new industrialization countries. Agriculture (irrigation, livestock, and aquaculture) is the largest consumer of water, it accounts for 69% of the world's annual water withdrawal. The next places are occupied by industry (19%) and household (12%) (World Water Assessment Programme, 2019). It is estimated that the global demand for water will continue to grow at the same pace until 2050, which will lead to an increase of 20–30% above the current level of water usage due to the development of the industrial and domestic sectors (Burek et al., 2016) of the emerging countries. Thus, the impact of human activities on the world state of water resources will further grow.

The problem of water pollution will become more and more important, turning it into one of the largest potential disasters for humanity in the coming century^1^. The irresponsible and ill-conceived approach to industry, urbanization, agriculture and environmental management, which humankind has adhered to in the recent past, the lack of adequate measures to prevent and eliminate polluting factors, as well as the lack and weakness of the mechanisms for bringing to justice the violators of environmental pollution, have led to the fact that water resources began to decline rapidly.

The UN General Assembly announced (Food and Agriculture Organization of the United Nations, 2011) that more than 1 billion (according to other estimates such as World Water Assessment Programme, 2019, more than 2 billions) people in the world suffer from a lack of safe water for drinking and household needs. Although the global average water deficit is only 11%, in 31 countries the water deficit ranges from 25% (the minimum threshold for water deficit) to 70%, and in 22 countries it exceeds 70% (United Nations Publications, 2018).

The main issues affecting the quality of natural waters (World Health Organization, 2017) include many different points:

- Infection with pathogens is an important factor in high morbidity and mortality from gastrointestinal diseases (Soprani et al., 2017). It is directly dependent on the population density and the level of its socio-economic development. Pollution by pathogens is not fully controlled even in developed countries.
- Contamination with organics (Cai et al., 2019), which enter the water in a dissolved or suspended form, mainly with sewage drains or unregulated household drains. Due to the oxygen dissolved in water coming from the atmosphere because of the turbulent nature of the flow, the rivers have a significant self-purification ability. However, when the supply of organics begins to exceed self-purification capability, water pollution progressively increases. Nowadays about 80% of polluted water resources are dumped back into oceans, rivers, and lakes^2^. In addition, the oxygen content in water is inversely proportional to its temperature; therefore, climatic conditions also play an unfavorable role in reducing the self-purification capability of rivers.
- Acidification is an anthropogenic natural process due to the increasing acidic reaction of the environment (Shi et al., 2016). It is accepted that natural waters are in a state of acidification if the pH is equal to or less than 5.0. Acidification is a consequence of dry and wet acidic deposition, the main components of which are aerosols consisting of sulfur and nitrogen oxides and ammonia, which, when interacting with water, form acids. This leads to a reduction or disappearance of crustacean, fish, insect, algae and zooplankton populations. The reproductive functions of aquatic organisms are also slowing down.
- Eutrophication enhances the biological productivity of water bodies due to the accumulation of biogenic elements in the water (Leaf, 2018). Excessive intake of nitrogen and phosphorus compounds (the main source of which is agriculture and household wastewater) leads to enhanced growth of aquatic plants, especially microscopic algae, which then result in the removal of large amounts of oxygen dissolved in water. This leads to negative consequences: reduction of fish populations, blocking of water intakes and spillways, deterioration of water quality.
- Agricultural fertilizers lead to an increase in the concentration of nitrates (Bouraoui and Grizzetti, 2014). Up to 15% of the initial mass of fertilizers goes into water bodies, mainly in groundwater. Excessive nitrate levels in drinking water can cause health problems, especially blood disorders in children and the risk of cancer in adults.
- Heavy metal pollution (Akpor et al., 2014): small but hazardous concentrations enter the global water supply from wastewater or from industrial waste landfills. Many heavy elements, such as lead, mercury, zinc, chromium, cobalt are toxic to both natural flora and fauna, and to humans.

As a consequence of the increasing risks and issues briefly described above, global water resources need careful control and monitoring. Preventing microbial and chemical contamination of a water source is the first stage of protection against contaminated drinking water and other public health concerns. For governmental and public environmental monitoring services, this task involves significant costs, the state-of-the-art hardware and software, and the work of highly qualified personnel who regularly maintain environmental monitoring tools.

Modern monitoring systems for water bodies consist of ground-based (stationary observations at hydrological and expeditionary posts) and remote (aviation and satellite) observation methods (World Health Organization, 2017; Sachse et al., 2018). They are also divided into contactless and contact observation methods; at the place of measurement—on portable, transportable, and laboratory; on data processing technology—on manual, automated and automatic procedures. In most cases, the information received from them is presented in a different format, even in terms of use within a single environmental organization, and is not integrated into a single information system (Hajdari, 2015).

Rapidity of data collection is of particular importance, especially for quick response to environmental changes in case of technical accidents and natural disasters whether they are or not of human origin (Wang et al., 2015). For example, oil can originate pollution episodes at all stages of production, transportation, processing until the final stages of consumption and disposal of related products. Tens of petroleum spills over 7 tons occur annually^3^, a lot of oil gets into the water due to leakage from pipelines, railways, oil-tankers, storage facilities.

Quick and cost effective ways of registering and logging pollution data are also needed, because in many cases it is necessary to determine the liabilities of the parties legally responsible for pollution episodes (Shimshack, 2014). For example, in most cases of noxious and unlawful waste disposal into waters produced by industrial enterprises or other entities, the analysis of water characteristics is performed manually by experts, often after complaints from citizens (Sebastian et al., 2018). The relative cost and lacking effectiveness of monitoring activities impair the processes of mitigation and control of human originated pollution.

One more example: the problem of eutrophication is often aggravated, among other things, by the unsatisfactory conditions of municipal and industrial wastewater treatment plants; in particular, this is especially true for reservoirs (Assemany et al., 2019). In order to monitor and control the purification infrastructures, it is necessary to monitor the content of nutrients causing abnormal coloration of waters, therefore an effective and reliable way to audit the infrastructure is required.

Another important issue is citizens' confidence in the monitoring systems of state and public environmental organizations (Alkhelaiwi and Grigoras, 2015). Official data, especially in developing countries, are often either insufficient or of dubious quality. Environmental experts point out that this is due to the obsolescence of the instrumental measurement base, the low financing of the environmental monitoring activities, political motives, and lobbying of the interests of polluting companies. The presence of a transparent system of monitoring the ecological state of water resources, in which the data obtained are verified and available for verification by every citizen, will raise the level of civil society engagement in the environmental conservation and contribute to reduce the skepticism about the need to finance this area and prevent the spread of environmental misconceptions (Arias et al., 2016).

As a consequence, an ideal system for monitoring the state of water resources should:

- Be cost-effective;
- Be small;
- Collect as much environmental data as possible;
- Have a high level of automation to minimize human influence;
- Be easily deployable, flexible, and scalable.

Today compact sensory systems are commercially available (for example, from companies like Libelium, Vaisala, Bosh) and are capable of measuring many physical and chemical indicators. They are able to provide researchers with quick results on environmental measurements, and such results are automatically sent to a secure data repository.

The work described in this paper explores the usage of a swarm of mobile platforms for the monitoring of the quality of water resources, capable of performing water quality measurements in automatic mode with minimal human participation (Shafi et al., 2018; Wang et al., 2018a,b). Autonomous water platforms, the so-called Unmanned Surface Vessels or Vehicles (USV), are nowadays hugely exploited, and many of them are commercially available, such as the PowerDolphin^4^, Texys Marine^5^, and CAT-Surveyor^6^ projects. These systems are small-sized, can be non-volatile and incomparably cheaper than the previous generation of equipment.

In this article, we describe the principles of certified collection of environmental samples using a small vessel equipped with sensors and connected to distributed registry for storing the collected data; such vessel leads a group of smaller USVs taking samples of the environment. We have performed our tests of the “leader” vessel at field, while the follower swarm behavior has been assessed by means of mixed reality simulation.

Robotics swarming, consisting in cooperative multi-agent autonomous systems, has a great potential in many field domains, and is especially suitable for marine environment monitoring, lending more flexibility and scalability to the overall system, as well as resulting in a greater effectiveness. Actual robotics swarming is not yet so widespread and exploited, due to many technical challenges that have to be addressed and solved, as very recently surveyed in Arnold et al. (2019)—even if such work is more focused on aerial vehicles, many concepts can be easily extended to other robotics application fields. This work highlights a set of features that a swarm must own, among which the ability to move, cooperate and/or react to occurring events. Another very recent work describes the deployment and exploitation of a heterogeneous robotic swarm for marine monitoring Lončar et al. (2019); however, authors present a multi-agent system with many different robots that have very limited motion capabilities: they adopt the concept of distributed communicating sensor networks, rather than implementing a robotic swarm as defined above (robots are mainly still in the neighborhood of their deployment points and do not cooperate to gather information). The topic of marine environmental monitoring by means of a robotics swarm is addressed also in Duarte et al. (2016), where the issue of scalability is faced through large-scale simulation. Finally, the issue of aggregation is addressed in a less recent work (Soysal and Sahin, 2005), proposing probabilistic aggregation strategies to obtain cooperative global behavior for the swarm, by combining basic individual behaviors. This approach traces back to the classical Reynolds flocking model described in Reynolds (1987), and here integrated in a Belief Space Planning strategy.

The core idea of the technological solution we propose is to merge a distributed ledger secure storage of the data with an effective sensor swarm. The goal is to have a shared control network system integrating a bio-inspired swarm management into a secure distributed ledger. Previously some authors have proposed either pure blockchain solutions (Kapitonov et al., 2019) or swarm solutions (Strobel et al., 2018) where each node is a blockchain node (something that makes the swarm operation slow).

We propose a hierarchical approach which is novel and puts together the benefits of both. The application of distributed ledger technologies in robotics applications is an emerging field, Castelló Ferrer (2018). A number of workshops have been organized in the latest few years, see footnotes^7^,^8^, and new publications are planned by the people working in this promising area, see footnote^9^. This work is related to two specific problems: the applications of blockchain technologies in swarm robotics and the application of blockchain technologies to networks robotics. In Strobel et al. (2018) the distributed ledger technology is used with the objective of guaranteeing the security of the swarm: the distributed secure ledger of the B-C ensure that “alien robots” do not join the network. This approach on the one hand allows to exploit the adaptivity of swarm intelligence and its capability to manage large numbers of robots, on the other hand applying blockchain algorithms to all the nodes in a swarm is at present difficult to put in practice for practical performance reasons. In Kapitonov et al. (2019) blockchain technologies are proposed as a tool to manage general ranging from Smart Cities to Citizen Science in multi vendor heterogeneous environments. However, the network robotics approach does not allow to efficiently and effectively manage a very large number of robots or intelligent devices and sensors.

In this work we do some steps to bridge the two approaches. Our platform shares the security features of distributed ledger technologies with the adaptivity and scalability of the swarm robotics approach. There are several benefits coming from the exploitation of a robotics swarm in a formation around the leader vessel:

- Many different measurements (of the same physical parameters) along the chosen path in only one mission. Having a cluster of measures around one point rather than just one is helpful in building up a measurement map more complete and reliable;
- Having more than one vehicle carrying sensors guarantees more robustness to the mission completion in terms of possible failures either of the vehicle or of the sensor;
- The follower USVs can be heterogeneous, i.e., equipped with sensors measuring different physical parameters in the current considered point along the chosen path.

Summarizing, a swarm operating in formation around the leader can provide more measurements (both in terms of quantity and in terms of different types), in a more reliable way, being equal the required mission time. The swarm approach provides higher levels of adaptivity and scalability with respect to other network robotics approaches with a limited pre programming and computational effort. Implementing it by means of an inherently stochastic planning motion method like BSP makes the solution comparatively robust. Moreover, in the foreseen overall system, the leader is the only one (having Internet access) in charge of storing the measures on the trustable platform; hence the vehicles around can share with it their gathered data, to be then integrated and aggregated by the leader to build up a map of the surveyed area.

This paper is organized as follows. Section 2 presents the idea of certified sampling based on distributed ledger technology and the related implementation based on the Robonomics platform. In section 3 the architecture and processes of such platform are detailed. Section 4 describes the “leader” vessel: its design, equipment, sensors, software, and the algorithms governing it. In section 5, the experimental results of water quality measurements obtained by the vessel are presented and analyzed. In section 6, we describe our Belief Space Planning implementation of Reynolds' Boids Model. Section 7 presents the results of the experimental tests of swarm behaviors in mixed reality simulation. Conclusions and discussion on future work are drawn in the last section.

## 2. Certified Sampling

The term “certified sampling” refers to the quality control of a biological or chemical sample for compliance with certain official criteria (Schreiber et al., 2006). The criteria, as a rule, are established by authorized legal entities or public authorities in the form of standards, regulations and sometimes laws, and the verification procedure itself is carried out by accredited specialists according to established rules and complying with established procedures. In theory, precisely this rigor of the sampling activities and the authority of the bodies performing them should foster public confidence in the obtained data. However, due to the high bureaucratization, the high cost of carrying out inspections and the concentration of control over them in the hands of legal bodies that are not always transparent, the public confidence in environmental data is decreasing (Jacques, 2016). In addition, the disclosure of serious corporate frauds (such as diesel emissions scandal^10^) have contributed to impair citizen trust in the “official” data.

The main requirements of certified sampling are standardized and registered execution procedures (on which all participants in the process agree) and confidence in the data received. The result of the audit is a formal certificate, whose format and content are legally defined, so that the change or forgery of the sample is often prosecuted as a crime. We propose a solution to reduce and mitigate the issues coming from bureaucracy, complexity and cost of inspections, as well as corruption.

The practical usage of mobile and stationary cyber-physical systems (CPS) that take environmental samples in automatic mode is growing (Mois et al., 2016). However, until recently, a general and reliable mechanism for automatically logging the actions of these devices was not proposed. Such a mechanism should not only save data, that device receives and sends. It should also guarantee their immutability and prevent collected data forgery. To this aim, we need to protect both the device executable code and their datasets (Bijani and Robertson, 2014).

In our setting the leader vessel acts as the centralization hub of the swarms samples and take care of the secure logging of the data by interacting with the Ethereum blockchain. The swarm collective behaviors are governed by our implementation of the BSP based flocking model. This allows to have at the same time a secure and certified log of the samples and an efficient management of the robot swarm, see 6. In this section we focus on the secure certification processes.

The foundations for the development of a distributed certification mechanisms have been actively studied by researchers and developers over the past decade: the blockchain technology. A blockchain provides a sequential chain of blocks built according to certain rules, and protected by cryptographic algorithms (Xu et al., 2016; Castelló Ferrer, 2018). The technology allows to create a peer-to-peer decentralized network of many nodes that exchange secure transactions. The main point of the technology is that it prevents data from being changed in transactions, but at the same time preserves the publicity of relations among the nodes in the network. Such a network of nodes will be protected from incorrect or malicious changes caused by a faulty data source, being this last either one of the nodes or an external attacker. Moreover, the blockchain allows to implement smart contracts—generated by a software program, placed on the blockchain with a guarantee of its implementation (Christidis and Devetsikiotis, 2016). Thanks to smart contracts, the operation of autonomous devices can be organized so that the program logic is executed only under the particular conditions specified in the transactions, and the data can be stored in an practically immutable registry.

The Ethereum blockchain (Dannen, 2017) smart contracts have been used to create the secure Robonomics platform^11^ and for the interaction of various autonomous devices. The central idea of the Robonomics platform is to organize the relationship between nodes in the form of offer and demand requests and to negotiate between them by using an internal currency.

The platform is based on a number of pillars:

- The Robot Operating System (ROS) (Koubaa, 2018). Due to the heterogeneity in term of system architectures and middleware of robotic systems and Internet of Things devices system interfaces and software, we used ROS to facilitate their interoperability and coordination. ROS makes it easier, in our case, to integrate new types of devices into a common network system architecture.
- The InterPlanetary File System (IPFS)^12^. We integrated IPFS into the platform to store the large amount of information that devices collect during their operations.
- Liability Market. This is the part of the platform in charge of matching Offer and Demand among the nodes of the system. The Liability Market transactions are organized through IPFS messages.
- Liability Contracts. They are Ethereum smart contracts made by cyber-physical systems (robots, intelligent agents, IoT devices and other artificial agents) with each other or with humans.
- Tokens. Since the interaction among the agents is based on market mechanisms, we need a “currency” in the network, and this is provided by “Tokens.”

The advantages of this approach for trustable measurements by USV are:

- This approach ensures that the data is collected and sent by specific USV.
- Once the data has been collected and sent to the network, it cannot be changed.
- The data remains open for verification by third party.
- There is an exact reference to the time when the digital signature of the data was sent to the distributed registry.
- Easy scaling of system. Thanks to integration with ROS, it's quite simple to add additional agents to the system, no matter what structure and mechanism of measurements.
- The unity of machine-to-machine and human-to-machine interaction in the context of ordering measurements.

These advantages enable the creation of a trusted communication environment which could potentially grow into a united ecological information system with a high level of trust. However, we should indicate the limitations of this approach:

- The need for internet access. IPFS and Ethereum require that the device always has access to these networks. The fourth generation mobile access network is sufficient for the stable operation of the system.
- Sending information to the blockchain requires monetary funds (since blockchain miners and some nodes of Robonomics network require a fee for their work).

It should also be noted that at the measurement stage, it is possible to physically intervene in the process. This problem can be solved by developing the proper USV design. For example, USV can record its activity in a photo or video and similarly save this information into Robonomics network.

In previous work of some of the authors, successful operation of several unmanned aerial vehicles on the Robonomics platform was demonstrated (Kapitonov et al., 2019); in the current research we are extending the approach to monitor the state of the environment, in particular of water quality. In this article we describe the operation of a small swarm of marine surface autonomous vehicles equipped with sensors in charge of performing water quality measuring, guided by a “leader” autonomous marine vessel managing a distributed secure registry of the samples by means of the Robonomics platform.

## 3. The Robonomics Platform

The platform is based on a middleware software called AIRA (Autonomous Intelligent Robot Agent)^13^ which enables the connection of ROS-based systems and devices with Ethereum and IPFS.

In general, following entities are required in the Robonomics network to complete a task:

- The Promisee, a node that assigns a task. This can be either a human or an artificial agent.
- The Promisor, a node that performs a task. It can be associated with physical or software tasks.
- The Liability Market, as mentioned above, is a platform for offer and demand messages published via IPFS.
- The Provider, a node that monitors the messages of the Liability Market and matches an offer and a demand for a small fee. The Providers of a “message channel” are managed by the “Lighthouse”—a special smart contract, which performs a transaction when the Provider establishes a market match between the Promisor and the Promisee.
- New liabilities in the form of smart contracts are concluded in the Ethereum network only via the Provider.
- The AIRA client is required for the Promisor to have access to the Liability Market and to get information about the task.
- The Validator (not shown), an optional node which may be specified in the demand message. If it is specified, only the Validator node (for a fee) can finalize liability contracts after checking them.

**Figure 1:**
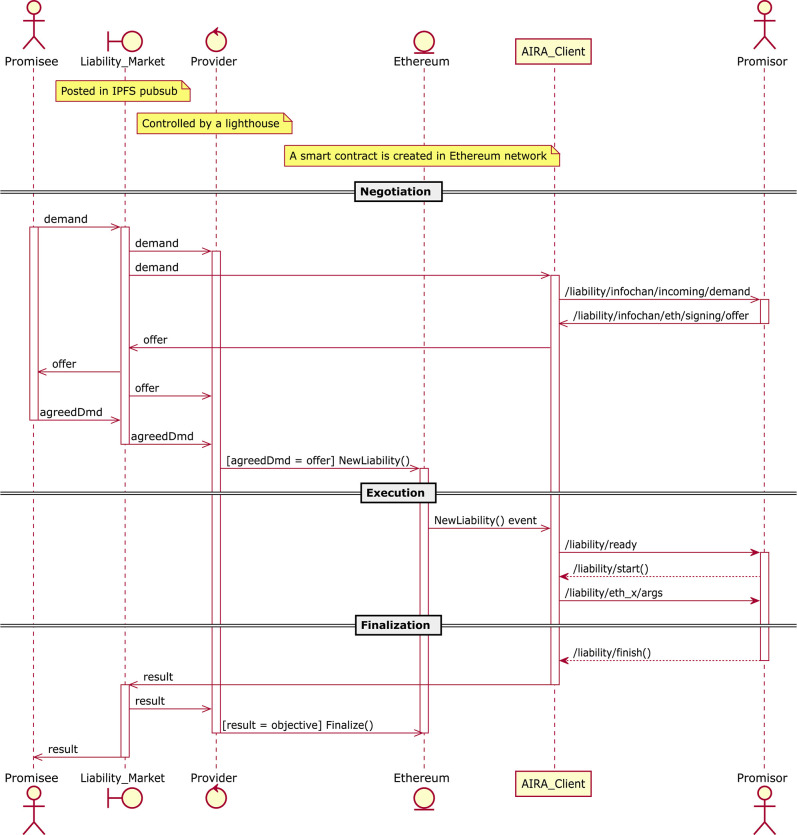
Nodes interaction in Robonomics network. Offer and demand messages between different nodes may vary in format (JSON in IPFS, ROS messages in AIRA client), but the information they provide remains the same.

The task performing process is organized in three stages, as depicted in Figure 1. At the first stage (Negotiation), the Promisee sends a demand message to the Liability Market in IPFS. In the following, the main message fields are reported (refer to Table 1).

**Table 1:** An example of a typical “Demand” message (without a Validator).

**Field**	**Type**	**Description**	**Example**
model	ipfs_common/Multihash	CPS behavioral model identifier	QmYb81uDNDHCnu9EZtYV 4eoBDKRBAwJeNy1LT3p5Zb c357
objective	ipfs_common/Multihash	CPS behavioral model parameters in rosbag file	Qmea8XkcSXmvLDKES7D88 6pfimsWh9Vjh1ZJsoHm9MW G4C
token	ethereum_common/Address	Operational token address	0xC02aaA39b223FE8D0A0e5 C4F27eAD9083C756Cc2
cost	ethereum_common/UInt256	CPS behavioral model implementation cost	0,1 WETH
lighthouse	ethereum_common/Address	Lighthouse address	0xa1b60ED40E5A68184b3ce4 f7bEf31521A57eD2dB1
validator	ethereum_common/Address	Validator address	0x000000000000000000000 0000000000000000000 (No)
validatorFee	ethereum_common/UInt256	Validator commission	0
deadline	ethereum_common/UInt256	Deadline block number	6393332
sender	ethereum_common/Address	Message sender address	0xB819d9BC2E665962BCa62 Cd859059875BABB134c
signature	std_msgs/UInt8[]	Sender's digital signature	—

*The offer message looks similar. Part of the fields is an IPFS hash on which significant information about the mission is located, another part refers to the node addresses in Ethereum network. Promisee exchanges such messages with Promisor until matching is reached between them. Between the layers, the messages change in the format (in IPFS it is JSON, in the AIRA client — ROS message), but the information remains the same*.

- The “model” field—it uniquely identifies the cyber-physical system.
- The “objective” field—it contains dynamic parameters specific for the operation to be performed (as arguments for functions in programming languages). This is an IPFS hash indicating the rosbag file. Such rosbag file contains ROS-topics and their details.
- The “token” field—the token used to pay for the service of the cyber-physical system.
- The “cost” field—the cost in tokens from previous “token” field.
- The “lighthouse” field—the name of the Lighthouse which manages the desired Providers.
- The “validator” field—the address of the Validator.
- The “validatorFee” field—validator fee for its work.
- The “deadline” field—block number until which the demand is valid.
- The “sender” and the “signature” fields—they are automatically filled and identify the Promisee.

The demand goes to the Provider, and then to the agent that is able to perform the task. CPS can accept the offer or submit a counter offer, in the same way, the Promisee can send counter-demand. This stage ends when offer/demand messages are equal in the *model, objective, token, cost*, and *lighthouse* fields. In this case, a new smart contract is created in the Ethereum Blockchain by the Provider.

When the Ethereum smart contract is created the requested task enters the Execution stage, during which the AIRA software waits for a message confirming the liability creation and passes the fields with the information to the agent. The CPS subscribes to the indicated ROS topics to obtain the necessary information; after this, the task execution begins.

In the last stage (Finalization), the CPS notifies the AIRA software of the completion of the requested task, and AIRA collects all the operation logs into the Result message. This message is then sent to the IPFS. If a Validator has been specified, it first checks the Result message and validate it. At the end the Provider sees the notification in the Result message and register the final transaction to Ethereum.

## 4. Description of the Autonomous Marine Vessels

The vessels are solar-powered water surface catamaran, with two hulls and a MPPT (Maximum Power Point Tracker) energy harvesting system with a lithium-ion battery.

Each vessel is equipped with:

- Two Bluerobotics T200 thrusters^14^;
- A waterproof case for electronics;
- A battery pack: LiFePO4 300 W·h, ∽2,000 charge cycles;
- Photovoltaic panels: 200 W max, 30-60 W while cloudy weather.

**Figure 2:**
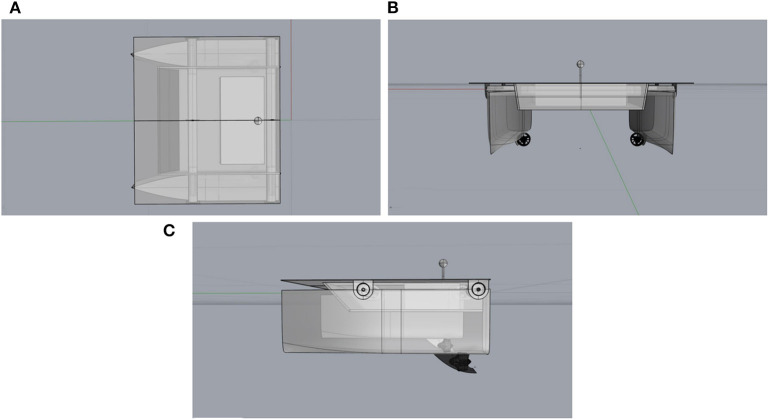
Top **(A)**, front **(B)**, and side **(C)** views of the main vessel.

The vessel main characteristics are listed in the following. Figure 2 illustrates its design.

- Max velocity: 5–7 km/h.
- Cruise velocity: 2–3 km/h.
- Dimensions: 1,200 × 1,200 × 500 mm.
- Weight: up to 38 kg (depending on the number of sensors).

Photovoltaic panels provide enough energy for daytime operations on cruise velocity, as well as 3–5 h of operations without light. The navigation and motion control system is based on Pixhawk and PX4 autopilot^15^. The choice of this controller is due to the fact that Pixhawk and PX4 are among the most popular tools for the navigation controller that natively supported operations with two motors without a steering device. The on-board computational unit is an Intel NUC; the water surface vessel has 2 slots for installing Libelium Waspmote Smart Water and Smart Water Ions platforms^16^.

Sensors are immersed with a winch. Such system is able to measure:

- pH
- Dissolved oxygen (DO), %
- Oxidation-reduction potential (ORP), mV
- Conductivity, μS/cm
- Temperature, °C
- Turbidity

Moreover, it is possible to detect ions presence, in particular: ammonium (NH4+), bromide (Br−), calcium (Ca2+), chloride (Cl−), cupric (Cu2+), fluoride (F−), iodide (I−), lithium (Li+), magnesium (Mg2+), nitrate (NO3−), nitrite (NO2−), perchlorate (CIO4−), potassium (K+), silver (Ag+), sodium (Na+).

In actual swarm configurations it is possible to integrate smaller and cheaper (sometimes expendable vessels) equipped with different and cheaper sensors.

### 4.1. Leader Vessel Hardware/Software Architecture

As told above, we plan to sample the environmental data by means of a small fleet of autonomous marine vessels. The “leader” vessel is in charge of the secure logging of the samples and leads the small swarm of vehicles performing the samples. In the following, we describe the “leader” vehicle on-board software. That software is based on the AIRA cyber-physical distribution kit running on Intel NUC. AIRA allows developers to implement “robotic tasks as a service” and allows the specification of a number of user parameters to customize the service itself. In the case of the vessel, the required “services” are the measuring and sampling missions. The user parameters of the mission are, in our case, the waypoints defining the vessel path and a list of sensors that should perform measurements during the robot motion along the requested path. The sensor samples are published via the IPFS network and are accessible by hash. This guarantees that the sampled data cannot be tampered and that they can be accessed by authorized persons (in our views the citizens, but in general different data accessibility schemes are possible).

The system software includes:

- General purpose Robonomics communication stack—standard set of components needed for connecting a CPS to the Robonomics platform:
  - Ethereum ROS API—vessel connection with the blockchain via ROS.
  - IPFS ROS API—vessel connection with IPFS via ROS.
  - Liability listener/Message signer—auxiliary services for liability: subscription to the Liability Markets, confirmation of the finalization of the liability.
- Application specific components:
  - Sensor data reader—reading and sending data from Libelium sensors.
  - Navigation package—motion control based on PX4 autopilot.
  - Trader node—the node that is responsible for possible “economic” behaviors (accept the request for measurements or not on the basis of an agreed price of the service). In our tests, the vessel accepts any orders, as we are exploiting the Ethereum distributed ledger as a secure ledger. In general, it is possible to describe any economic behavior (for example, in real world settings, there might be more vessel fleets offering the same service; agents may have to choose which are the most advantageous offers, etc.)

The measurement algorithm works as follows:

1. The user sends the demand for the execution of the measurement mission. The “model” field determines the type of service requested, while the parameters for its execution are transferred in the “objective” field. These parameters are added to the “objective” rosbag-file directly or in a string message containing an IPFS hash link. In our case, the waypoint file with a description of the path and stopping time at each of the points is transmitted in the ROS-topic “/waypoints” by a hash reference. The “/sensors” topic contains a line with a list of sensors, whose readings should be transmitted according to the result of the mission. The boolean topic “/virtual” contains the permission to perform the measurement mission virtually—to load the sensor readings as a result of the mission, measured previously at the points described in the “/waypoints” path. The “expiration” topic (Duration type) contains the expiration date of measurements for use in a virtual mission.
2. The demand is broadcast over the Robonomics network, AIRA software checks the parameters and sends an offer with the same parameters for order acceptance: model, objective, payment token, price and validator.
3. Both messages fall into the network and remain in the queues of providers of Robonomics. Matching offer and demand allows one of the providers to create a liability contract based on a delayed transaction mechanism^17^. The appearance of a contract in the blockchain, under which the vessel undertakes to execute a model with parameters from the “objective” field, confirms the appearance of an economically significant transaction (reservation of the customer's funds). This is a signal for the vessel to start working.
4. The vessel executes the contract: it loads the model and the objective from IPFS and starts the extraction of data from the “objective.” The navigation and motion planning system receives waypoints and stop time intervals for measuring data on each points. The measuring system receives a signal about which sensors should be turned on. During the work, the data is written to a file on the vessel's on-board computer.
5. When the last waypoint is reached and the measurement is taken, the measurement mission is completed. The archive with readings of water quality sensors and geodata are added to IPFS. IPFS hashes are written to the result file in the rosbag format. Its IPFS hash is sent in a transaction to a liability contract with a digital signature.

The distributed ledger implementation protects the monitoring data from counterfeiting or from the hiding the fact itself of having performed the measurements. The location of the hash of the measurement file signed by the private key of the robot in the automatically guaranteed repository (the blockchain) makes the verification of data authenticity simple: we just need to check the IPFS hash of the file and verify it with that recorded in the “result” field of the liability contract. If these hashes do not coincide, it is obvious that the robot received another counterfeited file.

The code of the vessel with Robonomics part is available here, in the footnote.^18^

The “follower” robots will implement the BSP algorithms in order to be able to follow the leader and to keep the formation.

## 5. Experiments and Analysis of Vessel Samples

In the field experiments, the marine vessel measured dissolved oxygen (%), temperature (°C), pH level, and electrical conductivity (μS/cm) in the surface water layer of the coastal part of the Volga river in Kuibyshev reservoir near the storm drains of Avtozavodsky district, Togliatti, Samara region, Russia. Sensor immersion depth: 1.5–2 m.

Date and time (local time — GMT+4):

- Beginning — 4/25/2019, 7:12:52 PM.
- Ending — 4/25/2019, 9:46:30 PM.
- Total: 154 min.

**Figure 3:**
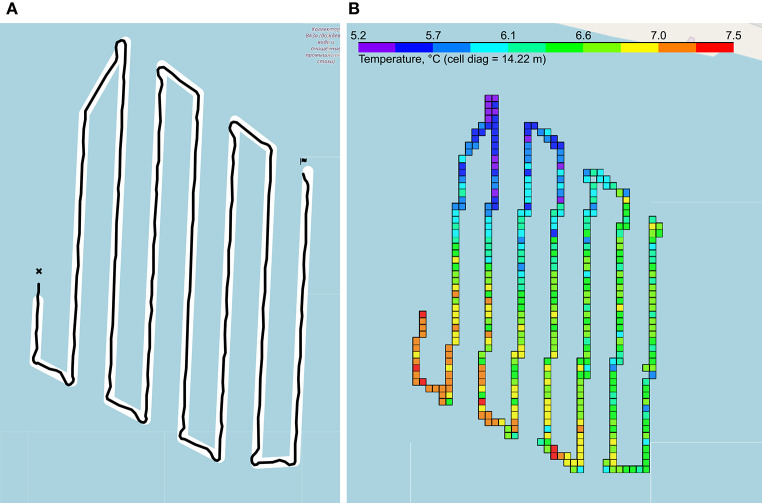
Measurement results. **(A)** Vessel traveled route: planned path in white, real path in black. **(B)** Example of temperature measurements: the colored cells show the averaged measurements of the temperature.

The route was set in the Ardupilot GUI^19^, which formed a file with waypoints. The planned and real routes are presented in Figure 3A.

As a result of measurements, the vessel sent data in the form of GPS and sensors data with Unix-timestamp. The Python programming language was chosen for working with data due to its ease of use, good performance and the presence of a wide range of libraries for file management, and data processing and visualization.

Measurements took place over long periods of time; they, together with GPS coordinates, were recorded discretely. Therefore, it was necessary to reduce such data into one structure, choosing from GPS only those coordinates that corresponded to the logs. The reconciliation was done using Unix timestamps. After extracting the data on the concentration of oxygen in water, it became clear that the representation of oxygen as a percentage is not enough for an adequate analysis of water quality, since in most cases it is necessary to translate the oxygen concentration in mg/l. Such a translation is non-trivial because it requires knowledge of the water temperature, normal oxygen concentration at normal atmospheric pressure at a given temperature, and atmospheric pressure in a given area. For that purpose, a dedicated software was developed. The data were visualized using the Folium Python library^20^, as in Figure 3B.

All obtained data and an interactive map with the measurement results is available in the footnote link^21^. Also the raw data that was sent to IPFS is available at the following links:

- Geodata: https://gateway.ipfs.io/ipfs/QmPvULEGfDE2Roscy4zGpKpBE8s3sBwjiXJVQNS3sBxWDC
- Measurement data from sensors: https://gateway.ipfs.io/ipfs/QmWRjFcQi4Xcisqi8FP3AbGS3PB3gNHgtnfzbcpodKKCBP.

### 5.1. Analysis of Environmental Data

In this subsection, we provide a short summary of the investigated reservoir collected data.

#### 5.1.1. pH Value

The concentration of hydrogen ions is of great importance for chemical and biological processes occurring in natural waters. In accordance with the requirements for the composition of water bodies in recreation areas and fishery reservoirs, the pH should not go beyond the range of 6.5–8.5. Based on the obtained data, the territory of the reservoir in terms of pH is more related to neutral and slightly alkaline waters and only a few segments are characterized by a high pH (alkaline waters with pH = 8.5 … 9.5).

#### 5.1.2. Electrical Conductivity

According to the electrical conductivity level of natural water, we can evaluate the mineralization of water. The conductivity in the studied area does not exceed the standards: the average value of the conductivity is 338.9 μS/cm, which corresponds to 169.45 mg/dm^3^ mineralization level. The studied water area can be attributed to the ultra fresh water category. It should be noted that electrical conductivity increases with distance from the coast. The conductivity results correspond to the regular dependence of electrical conductivity and temperature, with a correlation coefficient *r* = 0.77 at *p* = 0.05, which characterizes a strong positive coherence.

#### 5.1.3. Oxygen

For dissolved oxygen, World Health Organization does not offer any value for indications of its effect on health. However, a sharp decrease in the oxygen content in water indicates its chemical and/or biological pollution. In the obtained data, the amount of dissolved oxygen varies from 0 to 12.9 mg/dm^3^. During statistical processing, the data were divided into two groups: in the intervals [0;1.7] and [8.5;13.6] with an average value of 6.759 mg/dm^3^. The obtained intervals characterize the level of water pollution in the studied reservoir as dirty waters (interval [0;1.7]) and clean waters (interval [8.5;13.6]).

Since the content of oxygen dissolved in water depends on the temperature of the water and its mineralization, a pair correlation analysis was performed to determine the relationships: with a sample size of *n* = 1194, the critical value of the Pearson correlation coefficient *r*_*xy*_ = 0.06 at *p* = 0.05.

Accordingly, the values of the concentrations of oxygen dissolved in water have very weak dependence on temperature (correlation coefficient −0.091), a weak positive dependence on pH (correlation coefficient 0.156), and no dependence on conductivity.

## 6. Reynolds' Boids Swarm Implementation by Means of a Belief Space Planning Approach

Swarm behaviors were developed by following the approach proposed by some of the authors in Bonsignorio et al. (2019). Such work extended and applied the approach proposed in Platt et al. (2010) to robotic swarm control. Older work on BSP (Belief Space Planning) by those authors dealt with the trajectory planning and control of a robotic 3D-printed manipulator with deliberately poor joint accuracy and actuation with no joint feedback (Zereik et al., 2015), as well as in the motion planning of a marine companion robot for diver assistance and support (Zereik et al., 2014). Belief Space Planning methods allow to concurrently reduce the uncertainty (expressed by the state estimate measure covariance) and reach the desired state. Those features make them very suitable to perform tasks in unstructured environments characterized by significant measure noise. A trajectory in the “Belief Space” for a vehicle moves it from its current state (for example a given position/orientation), represented as a Gaussian PDF (Probability Density Function), to a goal state PDF with the desired mean value and lower covariance. The system state is modeled as the sum of a signal component and a Gaussian noise part.

**Figure 4:**
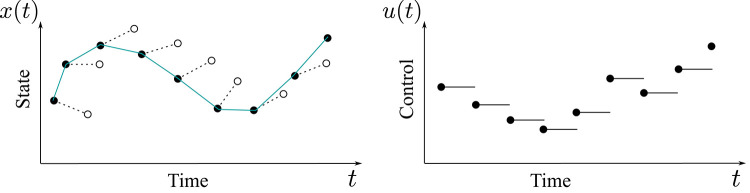
Direct Transcription is a special kind of Non-linear Programming Optimization method. The control variables are discretized as piecewise-constant trajectories while the state variables are represented by linear segments. The optimization is performed by considering the discrete values of the variables representing the control and the state at each segment endpoint.

The trajectory planned in the belief space for the vehicle is linearly approximated by a series of segments in the belief space. The initial and end points of each segment are determined by Direct Transcription. Such discretization method is depicted in Figure 4; for further details refer to (Platt et al., 2010) and (Betts, 2010).

The algorithm moves on the Belief Space trajectory piece-wise, segment by segment. The needed control actions to move from one segment extreme to the beginning of the following one are computed through a Linear Quadratic Regulator (LQR). The procedure is iterated on the segments of the linearized trajectory until the vehicle reaches its final goal in the belief space, represented by a vector of Gaussian PDFs with desired mean values for the end point and orientation, as well as a reduced covariance of the point and orientation expected measures. When a single vehicle follows a requested path, at each step, the current reference trajectory point is sent to the vehicle controller that drives the robot in the requested intermediate position. The final goal in the belief space is reached by iterating this process. At each step, the algorithm verifies if the current calculated intermediate end point of the computed segment of the plan is good enough to approach the final position and, if not, re–plans intermediate point. In our case, all companion autonomous vessels follow the trajectory of the leader vessel—the one managing the measure certification functions—by maintaining a predefined distance (some of them in terms of the cross-track error, some in terms of distance along the path). In this way, the whole robotics swarm system maintains a rhomboid formation while following the required trajectory. All the swarm vessels follow the leader, which is in charge of managing the certification of the samples by means of the distributed ledger processes provided by the blockchain platform.

Since all actions in the belief space actually weigh the objective to move the robot in the desired position with the objective of reducing the observation covariance in the mid waypoints and at the end point, they can be seen as information gathering actions (as they reduce the uncertainties on the vehicle position). The planning occurs in a state space which is inherently non-linear and has a higher number of dimensions than the physical state space; as a consequence the resulting dynamic is significantly underactuated (as the number of control input affecting the physical system is lower than the dimensions of the belief space). The problem can be simplified by the assumption of maximum likelihood of observations, as in the present paper, in our previous work and in Platt et al. (2010). This maximum likelihood assumption asserts that the current system state is the most likely state according to the past observations and the performed actions; this means that the performed actions achieve their intended purpose, leading the system to the desired state. Our simulations (in various application contexts) confirm that this assumption is usually correct. In Platt et al. (2010), a formal proof is provided about its optimality under linear Gaussian process assumptions. Here, the observations *z*_*t*_∈ℤ of the distance between the goal and the vehicle position are modeled as a non-linear stochastic function of the vector *x*_*t*_∈*X* , representing the (not directly observed) state of the system and of the environment

(1) $$\begin{array}{r} z_{t}=g\left(x_{t}\right)+\xi \end{array}$$

where *g* is a deterministic function of the measurement and ξ is a zero mean Gaussian noise with covariance *W*_*t*_, dependent on the state. The deterministic function *f* links the new state to the older one under the control action *u*_*t*_

(2) $$\begin{array}{r} x_{t+1}=f\left(x_{t},u_{t}\right) \end{array}$$

where *f* and *g* are assumed to be differentiable functions of *x*_*t*_ and *u*_*t*_. The controller is assumed to know the state through a probabilistic density function *P*(*x*). The parameters of such a distribution are the “belief state” $b_{t}={\left[m_{t}^{T}\;s_{t}^{T}\right]}^{T}$, where *m*_*t*_ is the mean of the belief state and $s={\left[s_{1}^{T},\cdots \;,s_{d}^{T}\right]}^{T}$ is a vector composed by the *d* columns of the covariance matrix Σ. If a linear Gaussian dynamics is assumed, the belief state can be updated by rules of the form

(3a) $$\begin{array}{r} x_{t+1}=A_{t}\left(x_{t}-m_{t}\right)+f\left(m_{t},u_{t}\right) \end{array}$$

(3b) $$\begin{array}{r} z_{t}=C_{t}\left(x_{t}-m_{t}\right)+g\left(f\left(m_{t},u_{t}\right)\right)+\xi \end{array}$$

where *A*_*t*_ and *C*_*t*_ are the Jacobian matrices $A_{t}=\frac{\delta f}{\delta x}\left(m_{t},u_{t}\right)$, $C_{t}=\frac{\delta g}{\delta x}\left(m_{t}\right)$. The Gaussian distribution is given by:

(4) $$\begin{array}{r} \Sigma_{t}:P\left(x\right)=N\left(x/m_{t},\Sigma_{t}\right) \end{array}$$

In these hypotheses, and assuming maximum likelihood of the observations, it can be proved that it is possible to derive by iteration a series of segments with an associated set of control actions by minimizing the cost function *J*

(5) $$\begin{array}{r} J\left(b_{\tau :T},u_{\tau :T}\right)=\overset{k}{\underset{i=1}{\sum }}{w_{i}\left({\hat{n}}_{i}^{T}\Sigma_{t}{\hat{n}}_{i}\right)}^{2}+\overset{T-1}{\underset{t=\tau }{\sum }}{\tilde{m}}_{t}^{T}Q{\hat{m}}_{t}+{ũ}_{t}^{T}R{ũ}_{t} \end{array}$$

where *b*_τ:*T*_ is the subset of the state space, *u*_τ:*T*_ are the corresponding actions for a given state space trajectory, *Q* and *R* are weight matrices, and the *n*_*i*_ are the versors of belief space along which the optimization is performed. Finally, Σ_*T*_ is the covariance matrix at the end of the segment, and $m_{t}^{T}$ the value of the mean of the Gaussian of the measures. The function *J* is minimized by a standard SQP (Sequential Quadratic Programming) algorithm; after this, a linear quadratic regulator is applied to move along the segments.

The procedure is summarized in Algorithm 1. The BSP strategy needs to know the initial belief state and final goal *b*_0_ and *b*_*goal*_, and returns the sequence *u*_1:*s*_ of the control actions. As a preliminary step all the variables of the algorithm are initialized to proper values via the function InitBSP (line 1 of Algorithm 1). The procedure is then executed for a predefined number of steps *N*. At each step, a plan is calculated via the CreatePlan function (line 3), obtaining the two sequences $\left({\bar{m}}_{1:s},{ū}_{1:s}\right)$. This plan is executed for *s* steps; in case the planned steps do not converge to the final goal, variable *k* and counter *r* (line 13) are in charge of managing the eventual re–planning. In this phase, three values are calculated (lines 6–8): *u*_*t*_ is returned by the LQR control, while *z*_*t*_ is the noisy perceived position measurement (see Equation 1); finally the value *m*_*t*+1_ is propagated through an Extended Kalman Filter (EKF). If the resulting error between the mean of the current reference belief state ${\bar{m}}_{t}$ and the mean of the current belief state *m*_*t*_ is under a given threshold *thr*_1_ (line 9), the algorithm sends the commands to the underlying vehicle low-level control system. The algorithm drives the robot, via the function DriveVehicle, toward the desired intermediate point in the trajectory (line 11), allowing the Cartesian error *e*_*t*_ to converge under a given threshold *thr*_2_ (line 10). The function DriveVehicle returns as output the necessary vehicle trajectories ***η***_*t*_. Finally, if the error $\left||{\bar{m}}_{t}-m_{t}|\right|$ is greater than *thr*_1_, the counter *r* is updated in order to proceed with a necessary re–planning step (line 13).

**Algorithm 1:** BSP Algorithm

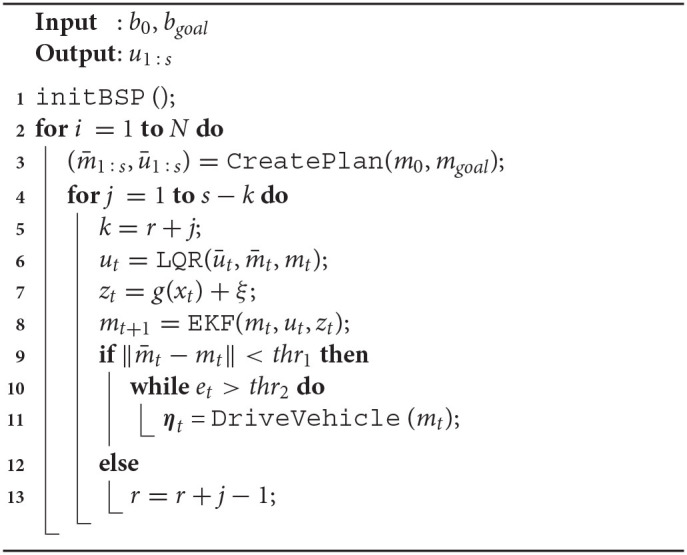

The adoption of LQR standard control improves the efficiency of BSP planning: the evaluation of the optimal control action for the system leads to the stabilization of the trajectory in spite of the non-linear dynamics of the system. Indeed, LQR control is able to handle small divergences from the planned motion, and thus minimizes the number of re–planning steps the system must calculate, improving in this way the computational efficiency. Previous experimental tests within a different application described in Zereik et al. (2015) showed that the statistical LQR calculation produces a decrease in computing time of about 70%. Hence, it is clear that the use of LQR in the algorithm leads to a smoother, and hence more efficient, motion of the robots.

The BSP approach is here integrated with the classical Reynolds flocking model. Reynolds has shown in Reynolds (1987) that flocking behaviors can be implemented by imposing to the individual agents a surprisingly simple set of rules:

1. *Separation*—Avoid crowding neighbors (short range repulsion).
2. *Alignment*—Steer toward average heading of neighbors.
3. *Cohesion*—Steer toward average position of neighbors (long range attraction).

In our system, agents target the school of (robot) fishes center (rules 2, 3) and keep distance among themselves (rule 1).

## 7. BSP-Based Swarm Mixed Reality Simulation

Using the BSP-based swarm approach described above, a swarm of vehicles has been simulated, with a different number *n* of companions, namely *n* = 4 and *n* = 8. The boat in charge of the sample collection and certification has been considered as the “leader” and the other vehicles have been requested to keep a rhomboid formation with the boat at the center, while following the master path. Note that the boat position at each instant is assumed to be known by the vehicle swarm. This is reasonable in a real application: indeed, the swarm and the boat move together along the reference path, so that the boat can easily communicate its current position to the vessel swarm, e.g., through a long-range WiFi or radio connection. Furthermore, each vehicle has its own GPS+IMU localization system on-board, in such a way to be able to determine its own relative position with respect to the main boat.

The tests of the swarm have been performed in a mixed reality simulation setting as the trajectory of the leader has been obtained by field tests of the vessel described above. We assume zero latency in the sample transmission from the follower vessels to the leader one. This assumption is realistic in comparison to the sampling rate and Ethereum typical transaction rate. The BSP swarm strategy is simple but very effective, since (as already stated) it allows each vehicle to follow the requested path while keeping the desired formation and, in the meantime, reducing uncertainty due to both inaccurate measurement and environmental disturbance. To this aim, in order to test robustness of the approach, beside the first simulation in nominal conditions, an additional Gaussian-distributed noise has been injected in the system, to stress the algorithm. In particular, the noise has been generated as a normal distribution with zero mean and covariance equal to *wW*_*i*_, where *w* is a tunable scalar coefficient and *W*_*i*_ is the corresponding diagonal component (*x*, *y* or ψ) of the covariance matrix *W*∈ℝ^3×3^ (each noise component is assumed to be independent and uncoupled from the other). For the angular component ψ, there is a further scale factor (equal to 0.1) to adjust noise values with respect to radians. Each diagonal element of matrix *W* is equal to $\sqrt{\underset{i}{\sum }{\left(\xi_{i}-\xi_{i}^{*}\right)}^{2}}$ (where ξ_*i*_ is the single component of the Cartesian error and $\xi_{i}^{*}$ is the related goal value for that component) if the Cartesian error is below a given threshold, or equal to a larger constant value *rg* otherwise.

For each formation type (*n* = 4 or *n* = 8 companion vehicles), two different values of *w* have been tested, namely *w* = 0.5 and *w* = 0.9; 10 simulation runs for each category were successfully executed, thus resulting in a total of 40 successful experiments.

**Figure 5:**
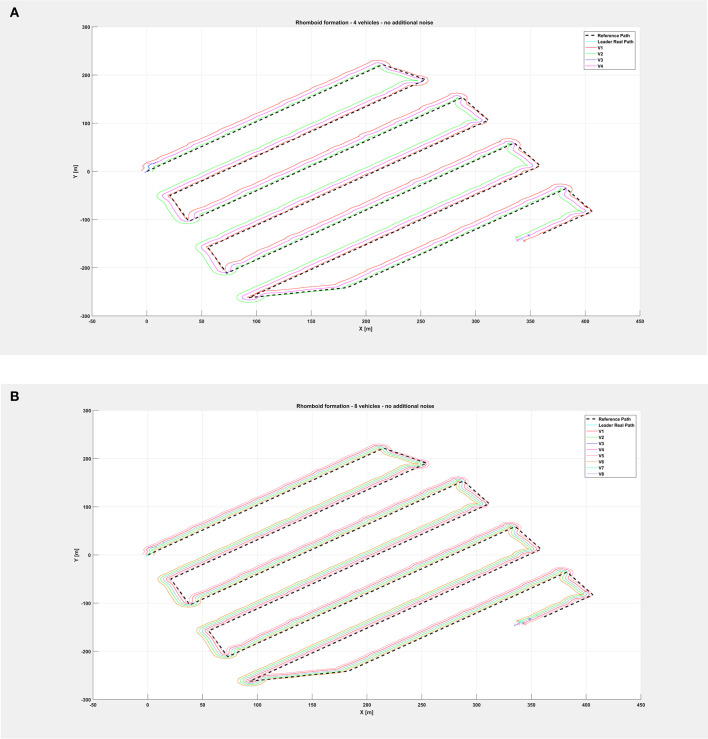
Overall path followed by the vehicle swarm in nominal conditions with **(A)** *n* = 4 companion robots, **(B)** *n* = 8 companion robots.

**Figure 6:**
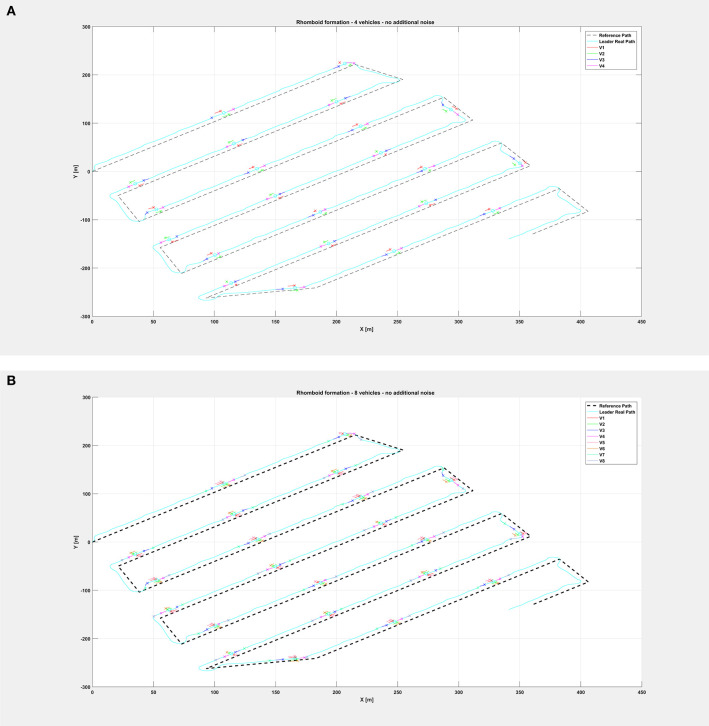
Overall path followed by the vehicle swarm in nominal conditions with **(A)** *n* = 4 companion robots, **(B)** *n* = 8 companion robots. Only selected time instants are depicted.

**Figure 7:**
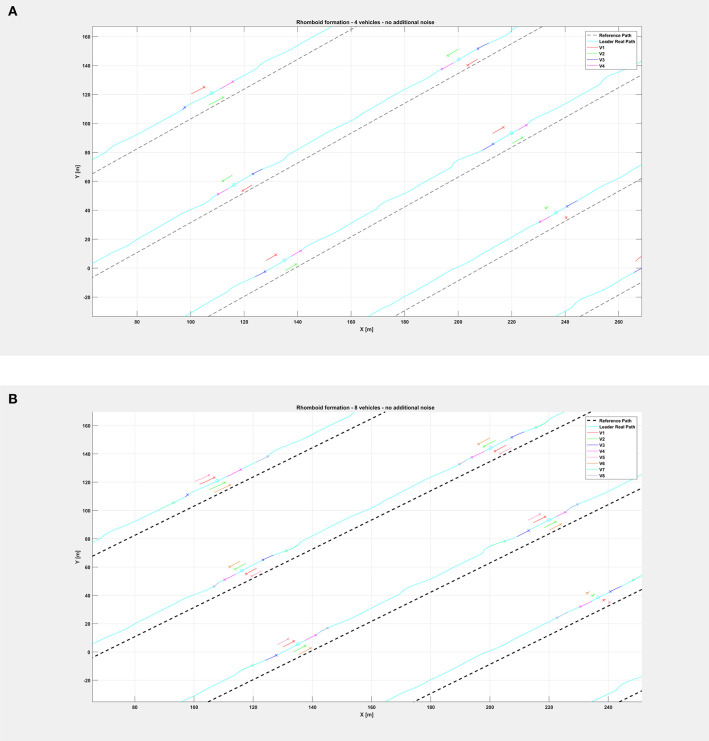
Insight of the formation at selected instants of the path followed by the vehicle swarm in nominal conditions **(A)** with *n* = 4 companion robots, **(B)** with *n* = 8 companion robots. Note the rhomboid and double rhomboid formations kept by the swarm in the two different cases.

Figure 5 shows the path followed by the vehicle swarm in nominal conditions (no additional noise injected in the system), while Figure 6 depicts only selected time instants of the path following execution, highlighting the rhomboid formation maintained by the swarm. Figure 7 provides a zoomed insight of a part of the simulation, showing the formation at some of the previous selected time instants.

**Figure 8:**
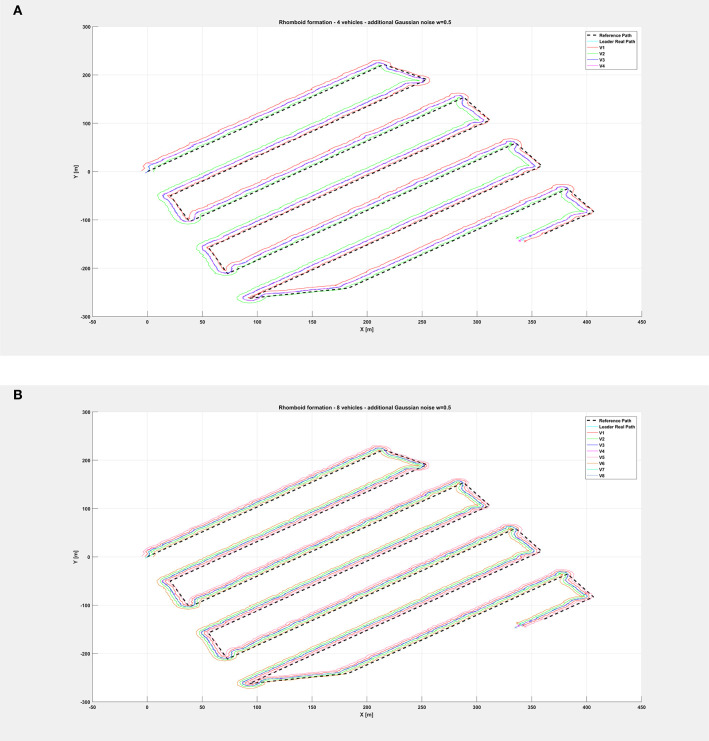
Overall path followed by the vehicle swarm with injected additional noise with *w* = 0.5 and **(A)** *n* = 4 companion robots, **(B)** *n* = 8 companion robots.

**Figure 9:**
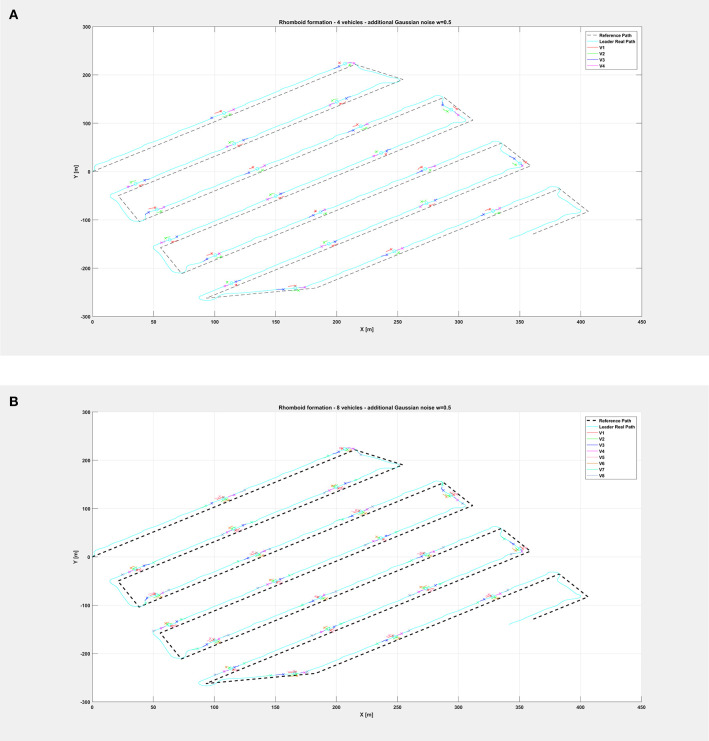
Overall path followed by the vehicle swarm with injected additional noise with *w* = 0.5 and **(A)** *n* = 4 companion robots, **(B)** *n* = 8 companion robots. Only selected time instants are depicted.

**Figure 10:**
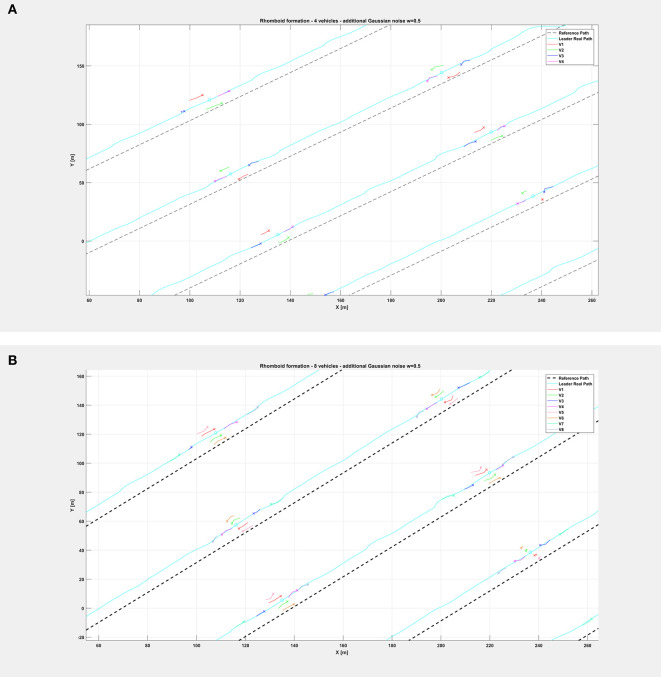
Insight of the formation at selected instants of the path followed by the vehicle swarm with injected additional noise with *w* = 0.5 and **(A)** *n* = 4 companion robots, **(B)** with *n* = 8 companion robots. Note the rhomboid and double rhomboid formations kept by the swarm in the two different cases.

The same organization is kept for the next figures: again, Figure 8 depicts the overall path followed by each vehicle when an additional noise with *w* = 0.5 is injected in the system, while Figure 9 shows only selected time instants of the path following execution, highlighting the rhomboid formation maintained by the swarm. Figure 10 provides a zoomed insight of a part of the simulation, showing the formation at some of the previous selected time instants.

**Figure 11:**
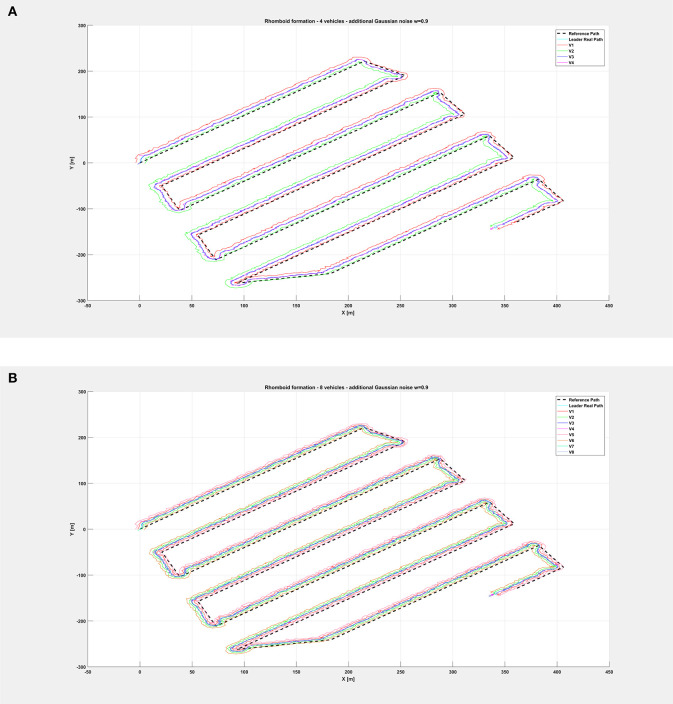
Overall path followed by the vehicle swarm with injected additional noise with *w* = 0.9 and **(A)** *n* = 4 companion robots, **(B)** *n* = 8 companion robots.

**Figure 12:**
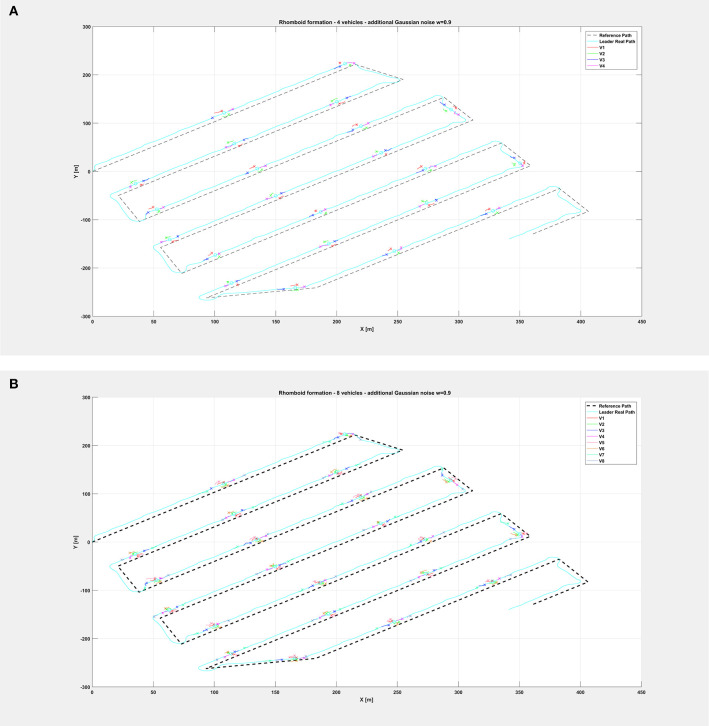
Overall path followed by the vehicle swarm with injected additional noise with *w* = 0.9 and **(A)** *n* = 4 companion robots, **(B)** *n* = 8 companion robots. Only selected time instants are depicted.

**Figure 13:**
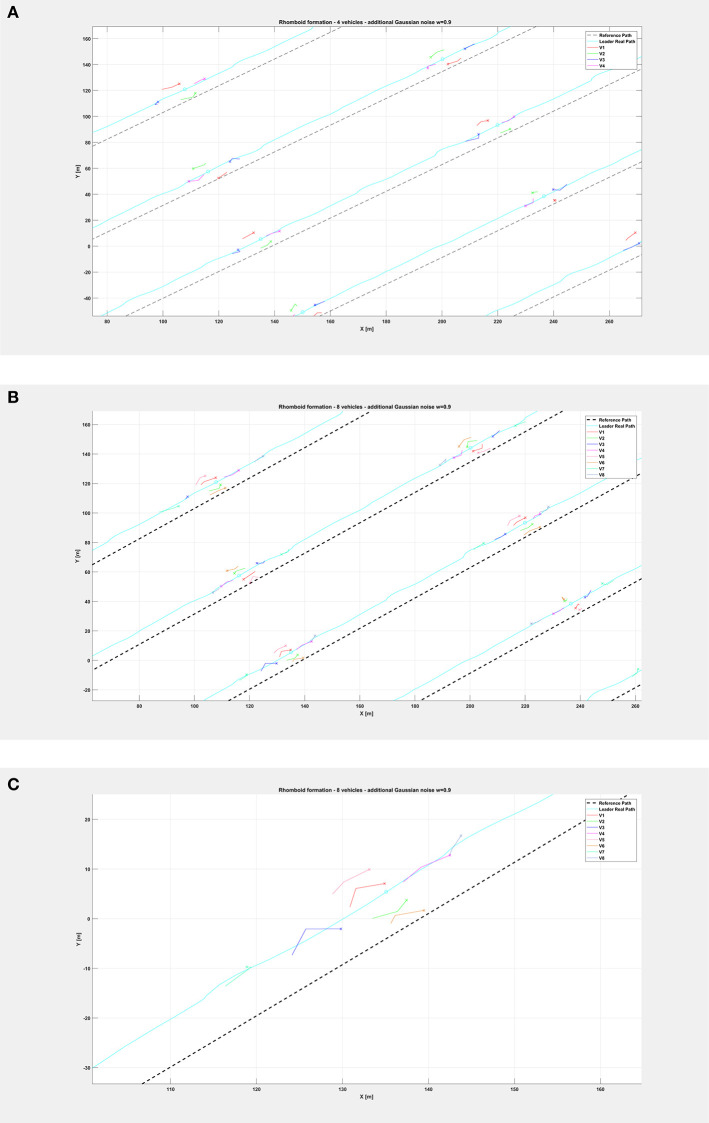
Insight of the formation at selected instants of the path followed by the vehicle swarm with injected additional noise with *w* = 0.9 and **(A)** *n* = 4 companion robots, **(B)** *n* = 8 companion robots (note the rhomboid and double rhomboid formations kept by the swarm in the two different cases). **(C)** Zoom of the fourth swarm formation (in the path order) of the previous **(B)**, highlighting that vehicles in the double rhomboid formation do not collide.

Finally, relatively to the case with additional noise with *w* = 0.9, Figure 11 depicts the overall paths followed by the vehicle swarm. Figure 12 shows only selected time instants of the path following execution, highlighting the rhomboid formation maintained by the swarm. Figures 13A,B provide a zoomed insight of a part of the simulation, showing the formation at some of the previous selected time instants. Figure 13C highlights a particular time instant of the simulation, in order to show that vehicles are not colliding.

**Figure 14:**
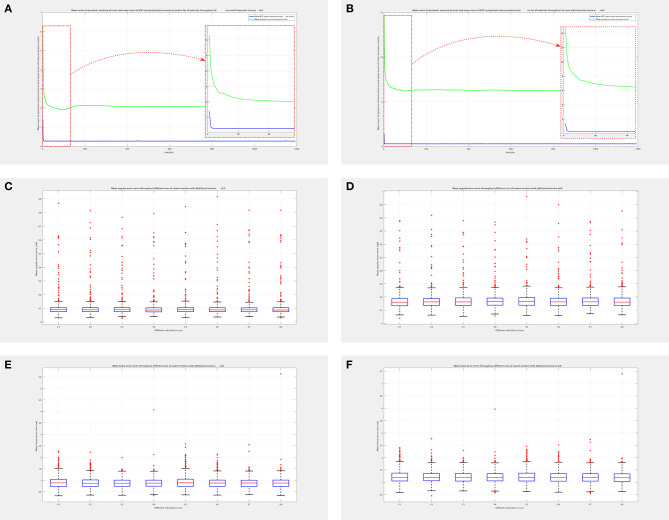
Simulation performance assessment for swarm with *n* = 8 companion vehicles. Mean norm of posterior variance of error and mean norm of BSP-computed state covariance matrix of all vehicles throughout all runs with noise **(A)** *w* = 0.5, **(B)** *w* = 0.9, with related zoom on the initial part of the graphs. Comparing norm of covariance matrix with that of posterior variance it is clear that the BSP strategy is able to quickly reduce the covariance matrix of system state, thus demonstrating its good performance. Mean angular error norm of all vehicles throughout all runs with noise **(C)** *w* = 0.5, and **(D)** *w* = 0.9. Mean linear error norm of all vehicles throughout all runs with noise **(E)** *w* = 0.5, and **(F)** *w* = 0.9. Please note that there are some outliers in the boxplots due to the fact that our approach provides a way-point control; the greater number of outliers in the angular component is due to the very steep angular changes in reference when the vehicles are required to invert their motion direction.

An assessment of good performance can be obtained by analyzing Figure 14. The evolution of mean norm of error posterior variance and of mean norm of the state covariance matrix Σ_*t*_, computed on-line by the BSP algorithm, can be compared from Figures 14A,B in both noisy cases. From this analysis, it is clear that the BSP strategy strongly reduces the covariance matrix on the system state and keeps it low during the whole simulation run, even if the Cartesian error is large. A zoom of the initial part of the graph is provided for both cases, for which the very fast reduction of the covariance matrix mean norm is clear. Boxplots of Figures 14C–F show the mean angular and linear error norm of all vehicles throughout all experiments. The related dataset can be found here: https://github.com/cyber-chicca/Swarm-BSP.

## 8. Conclusions and Future Work

We have developed a system that allows certified and trustable environmental sampling and logging by joining a sample certification scheme based on blockchain technologies and swarm behaviors based on a BSP implementation of Reynolds' Boids. Our experiments at field, integrated by extended mixed reality simulation, show the viability of the approach.

This article describes a new platform for trustable environmental monitoring based on citizen-led certification of the samples by means of distributed ledger technologies. This constitutes a further step in “Citizen Science.” The idea of citizen science, see Hippel (1991); Gura (2013); Hand (2010) is usually implemented by sharing with the public experimental data collected by one or more public or private organizations. In our case the citizens, thanks to intelligent robotics technologies integrated within a distributed ledger framework, have under their control the collection of the data themselves. This is particularly important for sensitive issues related to environmental quality, but can be relevant in many other societal relevant issues, for example the geographical distribution of infected people during a pandemic. We have shown that this conceptual approach can be implemented on top of the Ethereum blockchain network in a robust way and scalable way. Our platform allows to merge the benefits of distributed certification of the samples, made possible by the blockchain technology, with the adaptivity and scalability of swarm architecture. It separates the processes related to the sample certification, managed by the leader vessel, from the processes related to the optimization of sample collection performed by means of a potentially heterogeneous swarm of smaller vessels dedicated to the physical execution of the sampling activities. We have shown how mixed reality simulation can be a valuable tool for the design of specific system architectures for specific applications. Simulation technologies cannot substitute field experiments. However, they allow a greater and more systematic set of test runs than usually possible in the field. The experiments that we have performed in the field, where we have equipped the leader vessel with the set of sensors that in future implementations will be spread among the smaller vehicles, have shown the trustability of the certification of the samples. The purpose of the swarm simulation was to show that it is possible to implement the swarming behaviors that we have devised and provide guidance for future developments of the platform and its deployment in the field.

In the future, we will develop and perform two-ways mixed reality simulation in order to refine the system design and we will then proceed to the implementation of real world swarms at field.

Our approach is scalable since we can manage more swarms with different leader vessels. We will also consider the implementation of the BSP swarming approach to a fleet of leader vessels and other approaches based on Gaussian Processes and information gain. We will also consider the possible benefits of Deep Reinforcement Learning methodologies for the platform described in this paper.

## Data Availability Statement

All datasets generated for this study are included in the article/supplementary material.

## Author Contributions

All authors listed have made a substantial and intellectual contribution to the work, and approved it for publication.

## Conflict of Interest

FB was employed by the company Heron Robots. SL was employed by the company Airalab. VB was employed by the company M2M Economy, Inc. (“Merklebot”). The remaining authors declare that the research was conducted in the absence of any commercial or financial relationships that could be construed as a potential conflict of interest.

## Footnotes

^1^Woodford, C. (2019). *Water Pollution: An Introduction*. Available online at: https://www.explainthatstuff.com/waterpollution.html (accessed October 15, 2019).

^2^Denchak, M. (2018). Water Pollution: Everything You Need to Know. https://www.nrdc.org/stories/water-pollution-everything-you-need-know. [accessed October 15, 2019]

^3^https://www.statista.com/statistics/268553/number-of-oil-spills-by-oil-tankers-since-1970/

^4^https://www.powervision.me/en/product/powerdolphin

^5^https://www.texysmarine.com/

^6^https://www.subsea-tech.com/cat-surveyor/

^7^International Workshop on Blockchain Technologies for Robotic Systems (BCT4ROS'2019), https://researchers.pagesperso-orange.fr/bct4ros2019/.

^8^Proceedings of the First Symposium on Blockchain and Robotics, 2018, Ledger Journal, http://ledgerjournal.org/.

^9^Decentralized AI and Robotics Using Blockchain Technology, Frontiers Research Topic, https://www.frontiersin.org/research-topics/10378/decentralized-ai-and-robotics-using-blockchain-technology.

^10^Schiermeier, Q. (2015). The science behind the Volkswagen emissions scandal. Available online at: https://www.nature.com/news/the-science-behind-the-volkswagen-emissions-scandal-1.18426

^11^Lonshakov, S., Krupenkin A., Kapitonov, A., Radchenkoet, E., Khassanov, A., and Starostin, A. (2018). *The Robonomics Platform Has Been Designed for the Integration of Cyber Physical Systems into Human Economy*. Available online at: https://robonomics.network/robonomics_white_paper_en.pdf (accessed October 15, 2019).

^12^https://ipfs.io/

^13^https://github.com/airalab/aira

^14^https://bluerobotics.com/store/thrusters/t100-t200-thrusters/t200-thruster/

^15^https://pixhawk.org/

^16^Libelium (2018). *Drones, Sensors and Blockchain for Water Quality Control in the Volga River to Promote Trustworthy Data and Transparency*. Available online at: http://www.libelium.com/drones-sensors-and-blockchain-for-water-quality-control-in-the-volga-river-to-promote-trustworthy-data-and-transparency/ (accessed October 15, 2019).

^17^Krupenkin, A. (2018). *Delayed Transactions for Solidity* [Russian]. Available online at: https://github.com/akru/blog/blob/master/posts/2018-02-09-delayed-transaction.markdown (accessed October 15, 2019).

^18^Water Drone with Robonomics, GitHub repository, URL: https://github.com/Fingerling42/frontiers-vessel-code.

^19^http://ardupilot.org/

^20^https://python-visualization.github.io/folium/

^21^Marine vessel data processing, GitHub repository, URL: https://github.com/Fingerling42/frontiers-vessel-data-processing
